# A Ratiometric Fluorescent Probe Based on RhB Functionalized Tb-MOFs for the Continuous Visual Detection of Fe^3+^ and AA

**DOI:** 10.3390/molecules28155847

**Published:** 2023-08-03

**Authors:** Xin Jiang, Wenwei Li, Min Liu, Jie Yang, Mengjiao Liu, Daojiang Gao, Hongda Li, Zhanglei Ning

**Affiliations:** 1 College of Chemistry and Materials Science, Sichuan Normal University, Chengdu 610068, China; jiangxin20220706@163.com (X.J.); liwenwen202202@163.com (W.L.); liumin200009@163.com (M.L.); yangjie_deyouxiang@163.com (J.Y.); liumengjiao@sicnu.edu.cn (M.L.); daojianggao@sicnu.edu.cn (D.G.); 2 Key Laboratory of Treatment for Special Wastewater of Sichuan Province Higher Education System, Chengdu 610066, China; 3 Liuzhou Key Laboratory for New Energy Vehicle Power Lithium Battery, School of Electronic Engineering, Guangxi University of Science and Technology, Liuzhou 545006, China; hdli@gxust.edu.cn; 4 Sichuan Provincial Engineering Laboratory of Livestock Manure Treatment and Recycling, Sichuan Normal University, Chengdu 610068, China

**Keywords:** metal–organic framework, sensor, Fe^3+^, AA, fluorescence probe

## Abstract

In this study, a red-green dual-emitting fluorescent composite (RhB@MOFs) was constructed by introducing the red-emitting organic fluorescent dye rhodamine B (RhB) into metal-organic frameworks (Tb-MOFs). The sample can be used as a ratiometric fluorescent probe, which not only avoids errors caused by instrument and environmental instability but also has multiple applications in detection. The results indicated that the RhB@MOFs exhibited a turned-off response toward Fe^3+^ and a turned-on response for the continuous detection of ascorbic acid (AA). This ratiometric fluorescent probe possessed high sensitivity and excellent selectivity in the continuous determination of Fe^3+^ and AA. It is worth mentioning that remarkable fluorescence change could be clearly observed by the naked eye under a UV lamp, which is more convenient in applications. In addition, the mechanisms of Fe^3+^- and AA-induced fluorescence quench and recovery are discussed in detail. This ratiometric probe displayed outstanding recognition of heavy metal ions and biomolecules, providing potential applications for water quality monitoring and biomolecule determination.

## 1. Introduction

Ferric ion (Fe^3+^), as an inorganic transition metal ion, is widely distributed in the environment and biological materials [1,2,3]. As an essential trace element, Fe^3+^ plays an important part in biological processes, both clinically and environmentally, and Fe^3+^ can disrupt cellular homeostasis and lead to the development of various diseases. [4,5]. A deficiency in Fe^3+^ can lead to malnutrition, iron deficiency anemia (IDA) and stunted growth, while excess Fe^3+^ can lead to arthritis, liver damage, kidney failure, diabetes, neurodegenerative diseases (Parkinson’s, Alzheimer’s) and even cancer (lung cancer, liver cancer). Therefore, the determination of Fe^3+^ is key to the early diagnosis of these diseases. In addition, with the development of the economic society and chemical industry, Fe^3+^, as a common pollutant, can be released into our ecosystem through various industries and human activities, causing inevitable damage to human health and the ecological environment [6]. Therefore, sensitive detection of Fe^3+^ ions plays a vital role in various biological systems and industries.

Ascorbic acid (known as vitamin C, or AA for short) is one of a class of biomolecules that are widely found in fruits and vegetables as an organic small molecule, as well as an important antioxidant that plays a considerable role in the course of daily human life [7,8,9]. AA has a significant modulatory effect on neurotransmitters and enzymes of the central nervous system, with a reference range of 0.6 to 2.0 mg/dL in a seemingly healthy population [10,11,12]. Variable concentrations of ascorbic acid in biological fluids were found in clinical medical studies, where ascorbic acid is an indicator for assessing the exact amount of oxidative stress in human metabolism and plays a key role in maintaining physiological processes in humans and animals [13]. AA can be used to treat colds and physiological disorders. However, abnormalities in AA in humans can lead to associated disorders such as diarrhea, hyperacidity and kidney stones. Therefore, the analysis of AA is crucial for food safety and disease diagnosis [14].

A large number of sensors based on various designs and operating principles are being synthesized and improved to facilitate detection [15,16,17]. At present, some relevant detection methods have been developed for the detection of heavy metal ions and bio-organic substances, including electrochemistry [18,19], colorimetry [20,21], chemiluminescence [22], enzymology and capillary electrophoresis [8,23]. However, these methods have some drawbacks: low anti-interference, poor reagent stability, poor detection accuracy, many interfering factors and poor injection accuracy. Therefore, it is of great significance and urgency to explore a new chemical-sensing method to detect and sense metal ions and other detection substances with the naked eye. The fluorescence analysis method [24,25,26,27] is called “nondestructive testing” due to its nondestructive, highly sensitive, highly selective and rapid detection characteristics, which has attracted considerable attention in the field of testing and is a relatively popular testing method [28].

**Scheme 1:**
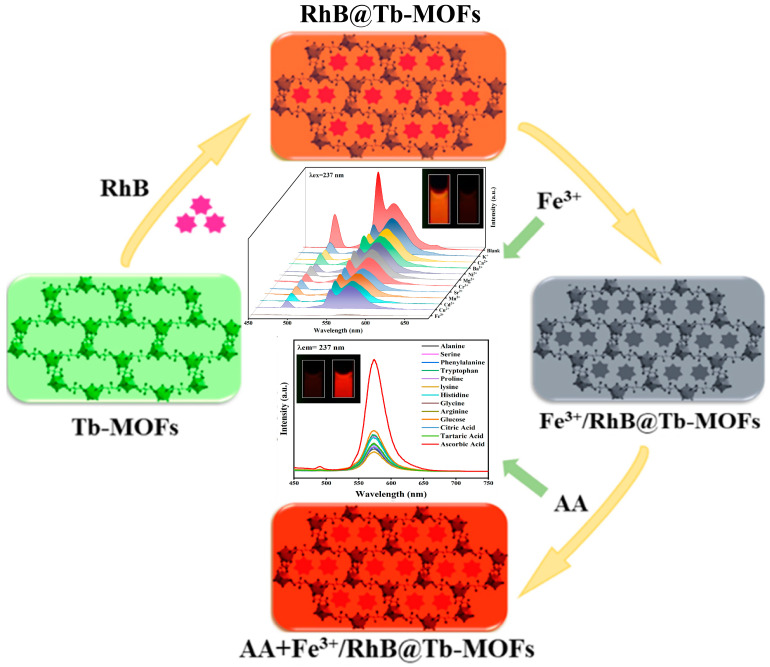
Schematic diagram of the RhB@Tb-MOFs for the continuous detection of Fe^3+^ and AA.

To address these issues, we designed a novel composite (RhB@Tb-MOFs), which was constructed by embedding fluorescent dye rhodamine B (RhB) in a porous microcrystalline Tb-based metal-organic frameworks (Tb-MOFs) and used as a novel fluorescence sensing platform (Scheme 1) for multi-domain detection (ion detection and biomolecule detection). When the sample RhB@Tb-MOFs were dispersed in an aqueous solution, the characteristic emission at 570 nm came from the RhB, resulting in improved photostability and extended fluorescence lifetime compared with free RhB. In addition, the RhB loaded inside and on the surface of the porous crystal could be used as the reference center so that the fluorescent probe has the ability for self-calibration and could well avoid environmental interference.

## 2. Results and Discussion

### 2.1. Characterization of the RhB@Tb-MOFs Fluorescent Probe

**Figure 1:**
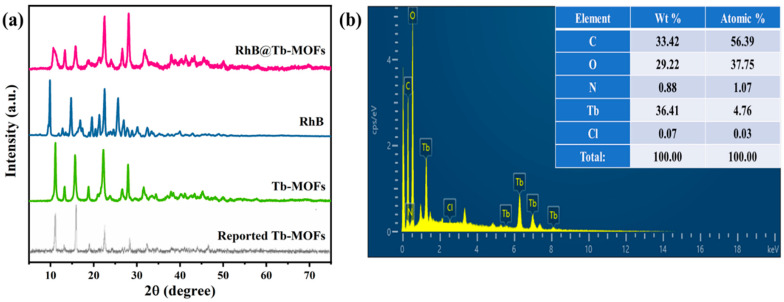
(**a**) XRD patterns of the reported Tb-MOFs, synthetic Tb-MOFs, RhB and RhB@Tb-MOFs (RhB: 0.44%); (**b**) EDX spectrum of the RhB@Tb-MOFs.

The XRD of the reported Tb-MOFs [29], synthetic Tb-MOFs, RhB and RhB@Tb-MOFs are shown in Figure 1a. The XRD of the synthetic Tb-MOFs was in good agreement with the reported Tb-MOFs. The XRD of the RhB@Tb-MOFs did not change significantly compared with the Tb-MOFs, indicating that the RhB modification did not damage the structure of the Tb-MOFs. The absence of the characteristic peaks for RhB in the XRD of the RhB@Tb-MOFs may have been caused by the low loading of the RhB in the MOFs matrix. In addition, elemental analysis of the RhB@Tb-MOFs is shown in Figure 1b. The RhB@Tb-MOFs contained five elements, namely, C, O, N, Tb and Cl, of which N and Cl are characteristic elements of RhB. Therefore, the chemical composition of the RhB@Tb-MOFs was consistent with that of the target sample, indicating that the composite was successfully prepared.

The emission spectra of different concentrations of the fluorescent dye rhodamine B (RhB) are shown in Figure S1a. As the concentration of RhB increased, the fluorescence intensity increased and then weakened due to its characteristic aggregation-induced quenching (ACQ) [30,31]. Therefore, the MOFs with the advantage of a porous structure were introduced to prevent aggregation quenching, thereby synthesizing dye@MOFs [31,32]. The emission spectra of the Tb-MOFs and the UV absorption spectra of RhB are shown in Figure S1b, where the spectra overlapped in the range of 500~600 nm, suggesting that there may have been an energy resonance transfer between the Tb-MOF and RhB that facilitated the interaction. RhB is red-emitting material and Tb-MOFs is a green-emitting material [33,34,35], and thus, may be prepared as red-green dual-emitting composites. Therefore, the functionalized modification of the Tb-MOFs with RhB in this study has corresponding theoretical support.

In order to accurately determine the content of RhB in the RhB@Tb-MOFs, the luminescence intensity of different concentrations of RhB was measured. As shown in Figure S2a, the content of RhB affected its luminescence and appeared red-shifted. The emission peak of RhB in the RhB@Tb-MOFs was located at 570 nm, which is within a reasonable range, indicating that the emission of RhB tended to be in the molecular state and the Tb-MOFs could effectively segregate RhB [1,36] to promote fluorescence emission. Figure S2b shows the linear fit between the concentration and intensity of RhB, yielding the relationship equation: *I* = 6.3033 × 10^8^ *C* + 240.4, where *C* indicates the concentration of RhB and *I* indicates the luminescence intensity of the RhB@Tb-MOFs. Thus, the specific content of RhB in the RhB@Tb-MOFs could be calculated using the relational equation.

The emission spectra of different concentrations of the RhB@Tb-MOFs are shown in Figure S2c. The luminescence intensity of the RhB@Tb-MOFs could be adjusted according to the variation in RhB concentration. Given that the RhB@Tb-MOFs acted as a ratiometric fluorescent probe, a RhB concentration of 1 × 10^−3^ M and a *I*_545_/*I*_570_ ratio of 1.5 were chosen as the target sample, and the actual loading of RhB was calculated to be 0.44% (low loading). The excitation wavelengths of the RhB@Tb-MOFs are explored as shown in Figure S2d. Different excitation wavelengths corresponded to different ratios of RhB@Tb-MOFs in the 225~245 nm range, and the excitation wavelength of 237 nm was chosen because the ratio of RhB@Tb-MOFs was guaranteed and verified it.

The IR spectra of RhB and RhB@Tb-MOFs are shown in Figure S3a. There was no significant difference in the IR of RhB@Tb-MOFs compared with the Tb-MOFs; this was probably because the RhB loading introduced into the pores and surfaces of the Tb-MOFs was too low to have an effect on the structure of the Tb-MOFs [37] (confirmed using XRD). The N_2_ adsorption test results for the Tb-MOFs and RhB@Tb-MOFs are shown in Figure S3b. The specific surface area and pore volume were 18.4238 m^2^/g and 0.0406 cm^3^/g, respectively, for the Tb-MOFs and 4.6310 m^2^/g and 0.0249 cm^3^/g, respectively, for the RhB@Tb-MOFs. By comparison, the specific surface area and pore volume of the RhB@Tb-MOFs were reduced by 74.9% and 38.7%, respectively, demonstrating that RhB was introduced into the pores or surfaces of the Tb-MOFs [38,39,40]. The thermogravimetric results for the Tb-MOFs and RhB@Tb-MOFs are shown in Figure S3c. The Tb-MOFs had three stages of weight loss (loss of water molecules, ligands, pyrolysis of the system); the RhB@Tb-MOFs had two stages of weight loss, namely, 5.7% at 200 °C and 16.1% in the 200~400 °C range, which were less than the weight loss of the Tb-MOFs in the same temperature range. The DTG curves of the Tb-MOFs and RhB@Tb-MOFs are shown in Figure S3d. The pyrolysis temperatures of Tb-MOFs were 249.6 °C, 352.8 °C and 462.8 °C, while for RhB@Tb-MOFs, they were 249.6 °C and 471.2 °C. The amount of pyrolysis of Tb-MOFs was the most obvious at 352.8 °C, while for the RhB@Tb-MOFs, it was smaller than that of Tb-MOFs at the same temperature. In addition, the pyrolysis process corresponded to the weight loss process, indicating that the RhB@Tb-MOFs had excellent pyrolysis resistance.

The SEM of the RhB@Tb-MOFs is shown in Figure S4a, in which a large number of spheres with diameters of about 3~5 μm were distributed, and the surface was relatively rough. The elemental mapping of the RhB@Tb-MOFs is shown in Figure S4b–f, and the five elements were uniformly distributed, which was consistent with the EDX test results, indicating the successful synthesis of the RhB@Tb-MOFs. The Tb-MOFs are shown as uniformly distributed circular spheres at high magnification in Figure S5a; the inset is a sphere with a smooth surface. The RhB@Tb-MOFs are also shown as uniform spheres in Figure S5b; the inset is a sphere with a rough surface (the surface attachment was RhB). The morphology of the RhB@Tb-MOFs did not change, indicating that the functionalized modification of Tb-MOFs by RhB did not affect the structure.

The UV absorption spectra of the RhB, Tb-MOFs and RhB@Tb-MOFs are shown in Figure S6. Compared with the Tb-MOFs (inset Figure S6b), the RhB@Tb-MOFs showed a distinct new absorption peak at 550 nm (inset Figure S6c), which is the typical UV absorption peak of RhB (inset Figure S6a), further indicating that the RhB@Tb-MOFs were successfully synthesized [41].

The XPS spectra of the Tb-MOFs and RhB@Tb-MOFs show the Tb 3d “splitting peak” of the Tb-MOFs and RhB@Tb-MOFs in Figure S7a. Figure S7b,c show a shift from 1242.5 eV (Tb-MOFs) to 1243.4 eV (RhB@Tb-MOFs), suggesting a weak interaction between the N of RhB and Tb^3+^. Figure S7d shows the O 1s peak shift from 531.5 eV (Figure S7e) to 532.5 eV (Figure S7f) in the Tb-MOFs and RhB@Tb-MOFs. The higher binding energy of the RhB@Tb-MOFs was probably due to the successful coordination of the free carboxyl group of the RhB to the Tb^3+^ of the Tb-MOFs, resulting in a decrease in the electron density of the O atom and an increase in the binding energy [42,43]. The above results confirmed the successful preparation of the RhB@Tb-MOFs [44].

### 2.2. Fluorescence Property of the RhB@Tb-MOFs Fluorescent Probe

**Figure 2:**
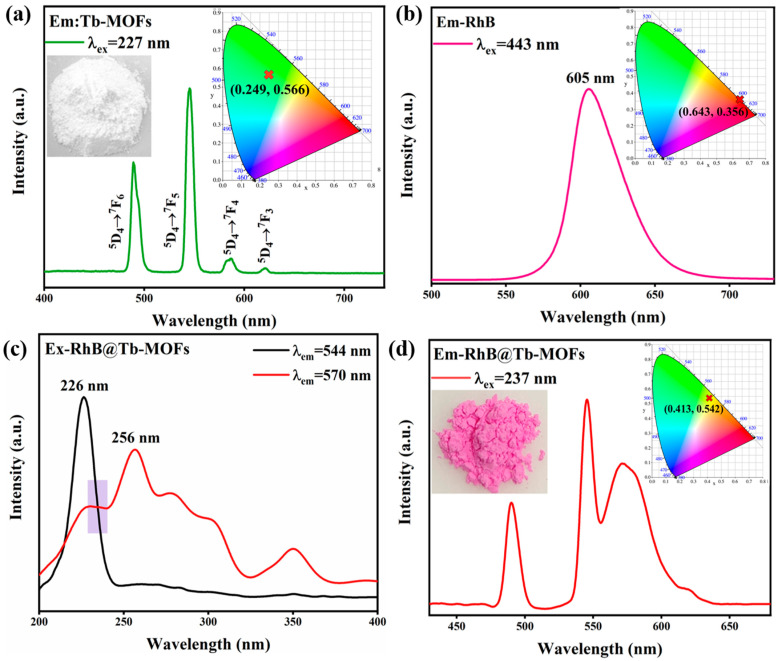
(**a**) Emission spectrum of the Tb-MOFs, where the insets are the photographs and the CIE chromaticity diagrams; (**b**) emission spectrum of the RhB, where inset is the corresponding CIE chromaticity diagrams; (**c**) excitation spectra of the RhB@Tb-MOFs; (**d**) emission spectra of the RhB@Tb-MOFs, where the insets are the photographs and the CIE.

The emission spectra of the Tb-MOFs are shown in Figure 2a. The CIE coordinate of (0.249, 0.566) is located in the green region, indicating that Tb-MOFs were green-emitting materials. As shown in Figure 2b, the RhB exhibited a characteristic red light emission peak centered at 605 nm, which is consistent with the reported RhB spectrum. The excitation spectra of the RhB@Tb-MOFs (Figure 2c) show ligand absorption and RhB absorption located at 226 nm and 256 nm, respectively, and these two peaks overlapped at 237 nm. The emission spectra of RhB@Tb-MOFs are shown in Figure 2d, which contains the characteristic emissions of Tb^3+^ and RhB (blue-shift due to concentration and solvation effects [45]), indicating that the RhB@Tb-MOFs were successfully prepared. The color of the powder changed from white (Tb-MOFs) to purplish red (RhB@Tb-MOFs). The corresponding CIE coordinate of the RhB@Tb-MOFs was (0.413, 0.542), which lies between the green and red regions, indicating that the RhB@Tb-MOFs belonged to the green-red dual-emission composites.

### 2.3. RhB@Tb-MOFs Fluorescent Probe Continuously Detected Fe^3+^ and AA

**Figure 3:**
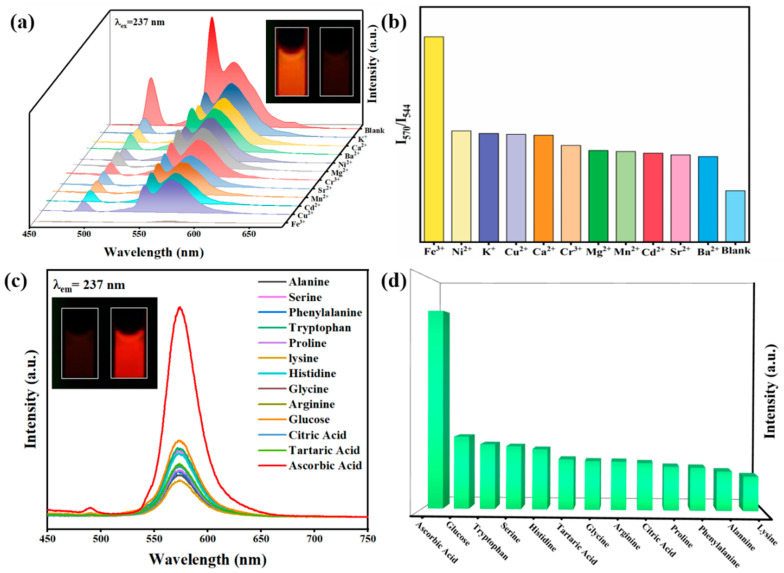
(**a**) Emission spectra of the RhB@Tb-MOFs added with different metal ions (10^−3^ M) in aqueous solution, where the inset shows the RhB@Tb-MOFs before and after detection of Fe^3+^ ions with 254 nm UV light; (**b**) *I*_570_/*I*_544_ of the RhB@Tb-MOFs containing different metal ions; (**c**) emission spectra of the Fe^3+^/RhB@Tb-MOFs added with different biomolecules (10^−3^ M), where the inset shows the Fe^3+^/RhB@Tb-MOFs before and after the detection of AA; (**d**) histogram of the luminescence intensity at 570 nm for the Fe^3+^/RhB@Tb-MOFs containing different biomolecules.

To investigate the ability of RhB@Tb-MOFs as fluorescent probes to detect metal ions, aqueous solutions of different metal ions (Fe^3+^, Ni^2+^, K^+^, Cu^2+^, Ca^2+^, Cr^3+^, Mg^2+^, Mn^2+^, Cd^2+^, Sr^2+^, Ba^2+^) were added to suspensions of RhB@Tb-MOFs. The results are shown in Figure 3a. The RhB@Tb-MOFs showed different fluorescence depending on which of the 11 metal ions was tested. By comparison, Fe^3+^ could remarkably quench the fluorescence of the RhB@Tb-MOFs. At the same time, the detection of the color change from orange-red to black could be directly observed with the naked eye. The histogram of the effect of the RhB@Tb-MOFs on the aqueous solution of different metal ions is intuitively presented in Figure 3b. The detection of Fe^3+^ was the most prominent and superior to other metal ions, indicating that the RhB@Tb-MOFs could selectively detect Fe^3+^. The anti-interference detection of the RhB@Tb-MOFs was also carried out. When Fe^3+^ was added to the RhB@Tb-MOFs suspensions treated with other metal ions, the emissions of the Tb^3+^ and RhB were almost quenched entirely (Figure S8a,b), similar to that with suspensions only treated with Fe^3+^, indicating that the RhB@Tb-MOFs were highly resistant to interference during the detection of Fe^3+^.

The sensing ability of Fe^3+^/RhB@Tb-MOFs for biomolecules was further investigated. Different solutions of biomolecules (alanine, serine, phenylalanine, tryptophan, proline, lysine, histidine, glycine, arginine, glucose, citric acid, tartaric acid and AA) were added to the Fe^3+^/RhB@Tb-MOFs and fluorescence tests were performed. As shown in Figure 3c, the RhB fluorescence of the Fe^3+^/RhB@Tb-MOFs was significantly restored after the addition of AA, and the results could also be directly distinguished by the naked eye. The histogram of the intensity (Figure 3d) shows the results of the Fe^3+^/RhB@Tb-MOFs for the detection of different biomolecules, with the best selective detection of AA. These results show that the RhB@Tb-MOFs could continuously detect Fe^3+^ and AA and had infinite potential as ratiometric fluorescent probes for the continuous visualization detection of metal ions and biomolecules. The influence of potential disruptors on the Fe^3+^/RhB@Tb-MOFs was studied (Figure S8c), where AA significantly restored the fluorescence intensity at 570 nm in the Fe^3+^/RhB@Tb-MOFs without interference from others. The results indicate that the Fe^3+^/RhB@Tb-MOFs had high selectivity and strong anti-interference for the detection of AA.

**Figure 4:**
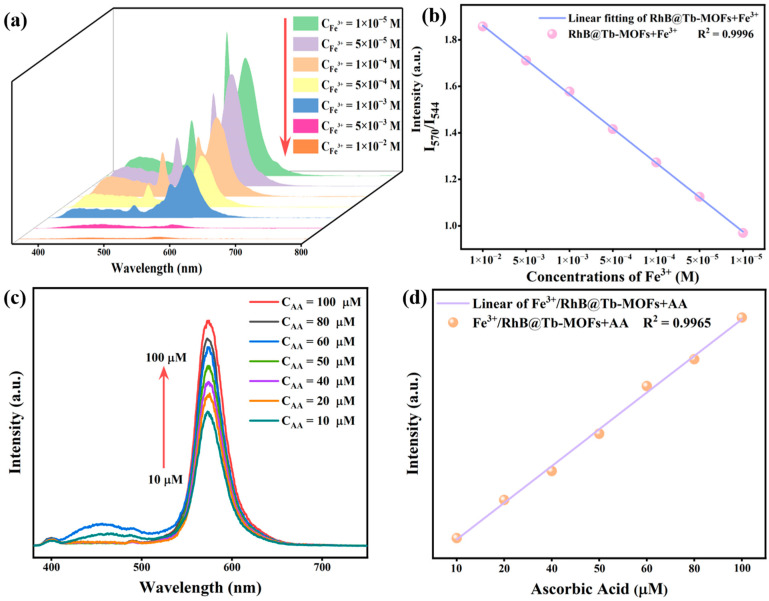
(**a**) The RhB@Tb-MOFs in response to different concentrations of Fe^3+^; (**b**) relationship between the *I*_570_/*I*_544_ of the RhB@Tb-MOFs and the Fe^3+^ concentration; (**c**) the Fe^3+^/RhB@Tb-MOFs in response to different concentrations of AA; (**d**) relationship between fluorescence intensity located at 570 nm and the AA concentration.

The quantitative detection capability of the RhB@Tb-MOFs was further explored. The effect of different concentrations of Fe^3+^ on the fluorescence intensity of the RhB@Tb-MOFs is displayed in Figure 4a. The fluorescence intensity gradually decreased with increasing Fe^3+^ concentration (10^−5^~10^−2^ M). A linear fit of the *I*_570_/*I*_544_ and Fe^3+^ concentration for this probe (Figure 4b) is given: Y = −0.15[M] + 2.01, where Y represents the *I*_570_/*I*_544_ of the RhB@Tb-MOFs and [M] is the Fe^3+^ concentration. The probe had a high linear correlation coefficient (R^2^ = 0.9996) and a low detection limit (LOD, 3σ/k) of 0.47 μM, indicating good quantitative detection of Fe^3+^ by the RhB@Tb-MOFs. Moreover, the fluorescence intensity of RhB in the Fe^3+^/RhB@Tb-MOFs gradually increased with increasing AA concentration (Figure 4c). The results were linearly fitted (Figure 4d) and show a good linear relationship in the 10~100 μM range, with the linear correlation coefficient R^2^ = 0.9965 and a detection limit as low as 2.54 μM. The above results indicate that the RhB@Tb-MOFs were highly sensitive as ratiometric fluorescent probes for the continuous quantitative detection of Fe^3+^ and AA. The results of similar probes for Fe^3+^ and AA detection limits are shown in Tables S1 and S2.

### 2.4. Detecting Mechanism

The structure of the RhB@Tb-MOFs, Fe^3+^/RhB@Tb-MOFs and AA+Fe^3+^/RhB@Tb-MOFs were tested using XRD and the results are displayed in Figure S9. The main diffraction peaks of the Fe^3+^/RhB@Tb-MOFs and AA+Fe^3+^/RhB@Tb-MOFs were not significantly different from the XRD of the RhB@Tb-MOFs. This result shows that the structures of RhB@Tb-MOFs remained relatively stable after detection, suggesting the mechanism of continuous detection of Fe^3+^ and AA by RhB@Tb-MOFs did not result in a change in structure.

**Figure 5:**
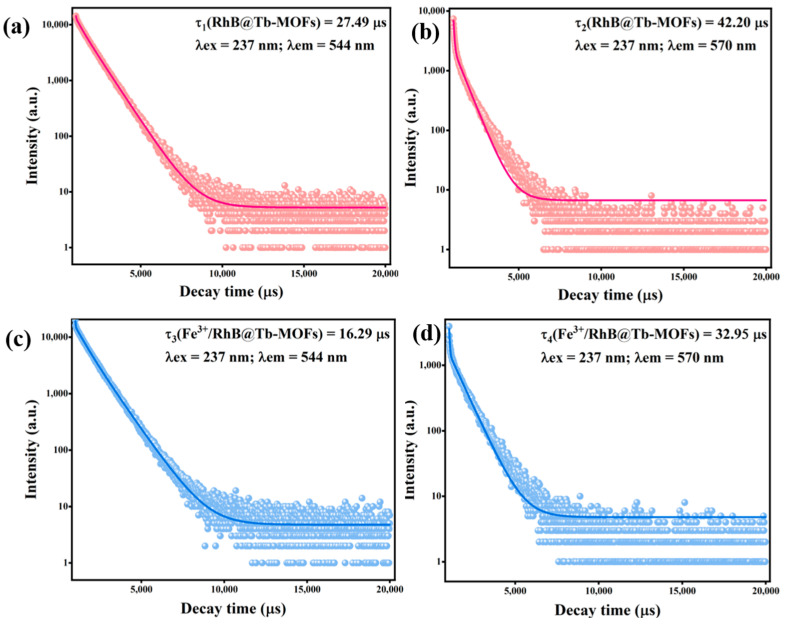
(**a**) Fluorescence lifetime of the RhB@Tb-MOFs at 544 nm; (**b**) fluorescence lifetime of the RhB@Tb-MOFs at 570 nm; (**c**) fluorescence lifetime of the Fe^3+^/RhB@Tb-MOFs at 544 nm; (**d**) fluorescence lifetime of the Fe^3+^/RhB@Tb-MOFs at 570 nm.

The fluorescence lifetime is another important verification to explore mechanism, the fluorescence lifetime tests were performed on the RhB@Tb-MOFs and Fe^3+^/RhB@Tb-MOFs. Figure 5a,b show the fluorescence lifetimes of the RhB@Tb-MOFs at 544 nm (τ_1_ = 27.49 μs) and 570 nm (τ_2_ = 42.20 μs). The fluorescence lifetimes of the Fe^3+^/RhB@Tb-MOFs were τ_3_ = 16.29 μs and τ_4_ = 32.95 μs at 544 nm and 570 nm (Figure 5c,d), respectively, both with reduced fluorescence lifetimes compared with the RhB@Tb-MOFs. The fluorescence lifetime was shortened due to the collision between the fluorophore and the quencher [46], indicating that the detection mechanism of the RhB@Tb-MOFs for Fe^3+^ was a dynamic quenching process.

**Figure 6:**
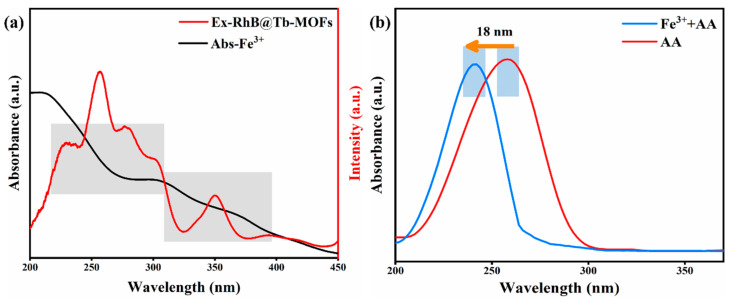
(**a**) Excitation spectra of the RhB@Tb-MOFs and the UV-Vis absorption spectra of Fe^3+^; (**b**) the UV-Vis absorption spectra of the AA solution and mixed solution of Fe^3+^ and AA.

The internal filtration effect (IFE) is one of the mechanisms of fluorescence quenching and has been widely used in biochemical assays. As shown in Figure 6a, the excitation spectra of RhB@Tb-MOFs and the UV absorption spectra of Fe^3+^ were tested, which overlapped in the short-wavelength range of 200~400 nm, and the energy transferred from the matrix to the luminescence center was absorbed by Fe^3+^, suggesting that the detection of Fe^3+^ fluorescence quenching was likely due to the IFE [44,47]. In addition, the mechanism of the fluorescence recovery of RhB after the detection of AA by the Fe^3+^/RhB@Tb-MOFs was investigated by testing the UV-Vis absorption spectra of the AA solutions and a mixture of AA+Fe^3+^. As shown in Figure 6b, the UV-Vis absorption spectrum of the mixed AA+Fe^3+^ solution was blue-shifted by 18 nm toward the shortwave wavelength compared with the AA solution, indicating a correlation reaction between the Fe^3+^ and AA, which ultimately led to the recovery of fluorescence of the RhB [1].

**Figure 7:**
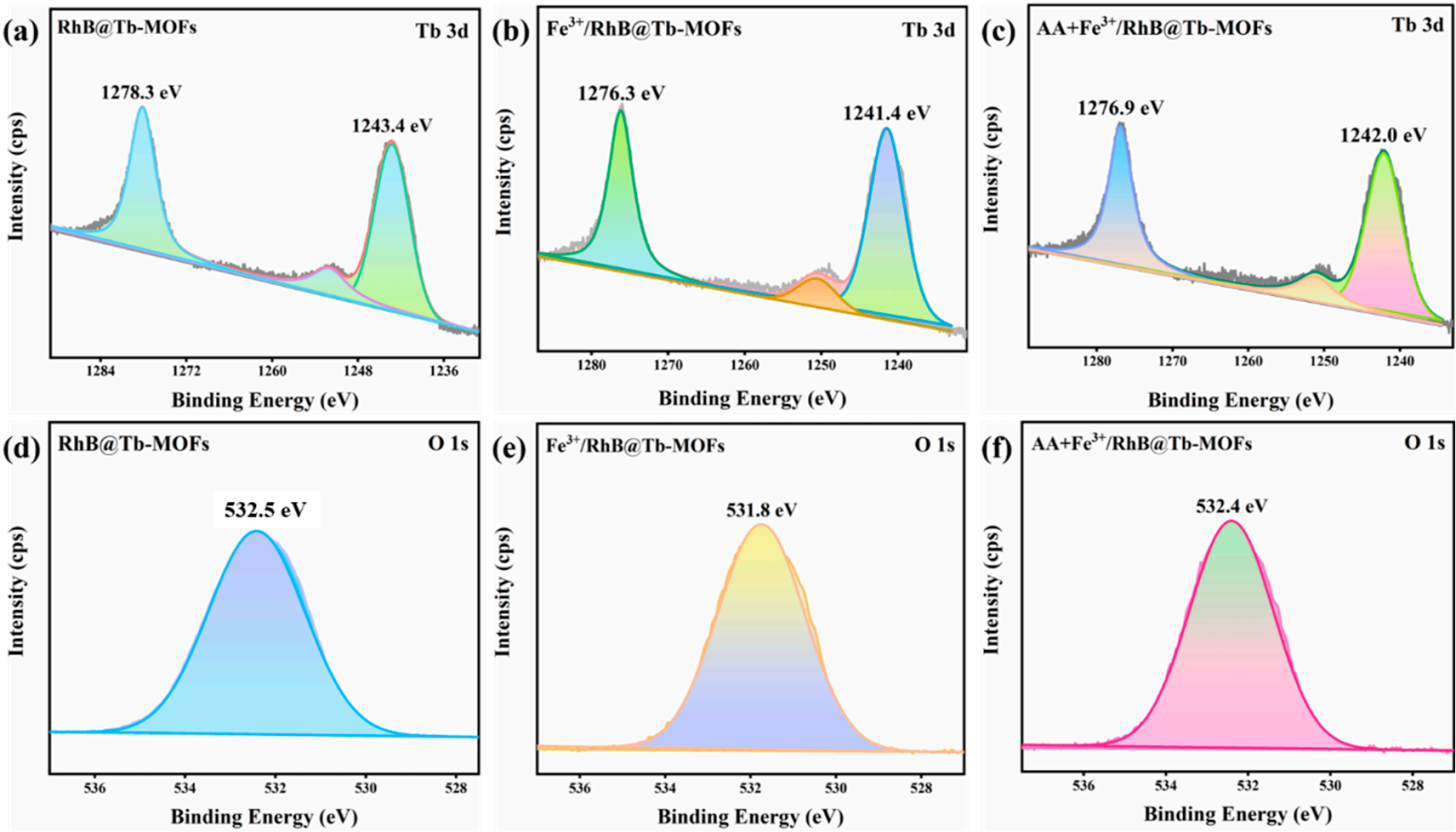
(**a**) The XPS peaks of Tb 3d in the RhB@Tb-MOFs; (**b**) the XPS peaks of Tb 3d in the Fe^3+^/RhB@Tb-MOFs; (**c**) the XPS peaks of Tb 3d in the AA+Fe^3+^/RhB@Tb-MOFs; (**d**) the XPS peaks of O 1s in the RhB@Tb-MOFs; (**e**) the XPS peaks of O 1s in the Fe^3+^/RhB@Tb-MOFs; (**f**) the XPS peaks of O 1s in the AA+Fe^3+^/RhB@Tb-MOFs.

In order to further explain the internal mechanism of the RhB@Tb-MOFs fluorescent probe for the continuous detection of Fe^3+^ and AA, XPS analysis was performed. Figure S10 shows the peak XPS spectra of the RhB@Tb-MOFs, Fe^3+^/RhB@Tb-MOFs and AA+Fe^3+^/RhB@Tb-MOFs (Tb 3d, O 1s and C 1s). To further analyze the changes in orbital valence, the “peak splitting” of Tb 3d and O 1s was tested. As shown in Figure 7a–c, the binding energy of Tb 3d at 1278.3 eV in the RhB@Tb-MOFs was shifted to 1276.3 eV in the Fe^3+^/RhB@Tb-MOFs, suggesting that Fe^3+^ may have interacted with Tb^3+^ in the RhB@Tb-MOFs and led to the fluorescence quenching. The binding energy of Tb 3d in the AA+Fe^3+^/RhB@Tb-MOFs was shifted to 1276.9 eV, with a partial fluorescence recovery of RhB@Tb-MOFs as a result of the interaction of Fe^3+^ with AA. As shown in Figure 7d–f, the binding energy of the O 1s peak in the Fe^3+^/RhB@Tb-MOFs shifted about 0.7 eV compared with that of the RhB@Tb-MOFs (from 532.5 eV to 531.8 eV), suggesting that Fe^3+^ may have caused a change in the O 1s site in the RhB@Tb-MOFs. The binding energy of O 1s of the AA+Fe^3+^/RhB@Tb-MOFs was also shifted 0.6 eV compared with the Fe^3+^/RhB@Tb-MOFs (from 531.8 eV to 532.4 eV). This may have been due to the presence of several oxygen-containing groups in the structure of AA, where it is likely that these O sites interacted with Fe^3+^ in some way, promoting partial fluorescence recovery of the Fe^3+^/RhB@Tb-MOFs.

## 3. Experimental Section

### 3.1. Reagents and Instruments

All of the chemicals and solvents used in this work were commercially available in analytical grade and were used directly. Terbium nitrate hexahydrate (Tb(NO_3_)_3_**^.^**6H_2_O), mucic acid (MA), Potassium hydroxide (KOH) and Rhodamine B (RhB) were obtained from Aladdin Chemistry Co., Ltd. (Shanghai, China). Sodium chloride (NaCl), Nickel chloride (NiCl_2_), Copper chloride (CuCl_2_), Calcium chloride (CaCl_2_), Chromium chloride (CrCl_3_), Magnesium chloride (MgCl_2_), Manganese chloride (MnCl_2_), Cadmium chloride (CdCl_2_), Strontium chloride (SrCl_2_), Barium chloride (BaCl_2_) and Ferric trichloride (FeCl_3_) were purchased from Chengdu Kelong Chemical Co., Ltd. (Chengdu, China). Alanine (C₃H₇NO₂), Serine (C₃H₇NO₃), Phenylalanine (C₉H_11_NO₂), Tryptophan (C_11_H_12_N₂O₂), Proline (C₅H₉NO₂), Lysine (C_6_H_14_N_2_O_2_), Histidine (C₆H₉N₃O₂), Glycine (C₂H₅NO₂), Arginine (C₆H_14_N₄O₂), Glucose (C₆H_12_O_6_), Citric Acid (C₆H₈O₇), Tartaric Acid (C₄H₆O₆) and Ascorbic acid (C_6_H_8_O_6_, AA) were purchased from Shanghai Bide Pharmaceutical Technology Co (Shanghai, China).

The detailed characterization is given in the Supplementary Materials.

### 3.2. Synthesis of the Tb-MOFs and RhB@Tb-MOFs

The Tb-MOFs sample was synthesized using a fast and facile method at room temperature [48]. The RhB@Tb-MOFs sample was synthesized by using a simple ultrasonic immersion method. First, the 50 mg products of the Tb-MOFs, 5 mL of 1 mM ethanol solution of RhB was added into the 10 mL centrifuge tube, and then the mixture was shaken uniformly and equilibrated evenly for 30 min using an ultrasound treatment. Then, it was soaked for 48 h. Finally, the precipitates obtained were collected centrifugally and dried in an oven for 24 h.

### 3.3. Fluorescence Sensing for Detecting Fe^3+^ and AA

In a typical operation, 3.0 mg of the RhB@Tb-MOFs sample powder was dispersed in a 10 mL centrifuge tube and ultrasound was performed for 10 min to obtain a standard stock solution of RhB@Tb-MOFs. Then, 5 mL of the RhB@Tb-MOFs original solution was mixed with 1 mL of Fe^3+^ solution with different concentrations (10^−2^–10^−5^ M). The resulting solution was thoroughly mixed and its fluorescence emission spectra were measured under 237 nm excitation. In order to determine the concentration of ascorbic acid, 1 mL of Fe^3+^ (10^−3^ M) was introduced into the standard stock solution of the RhB@Tb-MOFs to form a sensitive and selective fluorescence sensing platform Fe^3+^/RhB@Tb-MOFs. Then, 1 mL of ascorbic acid with different concentrations (10–100 μM) was added, the mixture was shaken and incubated at room temperature for 30 min, and the fluorescence spectrum was measured.

## 4. Conclusions

In summary, a ratiometric fluorescent probe, namely, RhB@Tb-MOFs, with red-green dual emission was prepared and designed for excellent continuous sensing analysis of Fe^3+^ and AA. The probe not only had the advantages of good selectivity, high sensitivity and high resistance to interference but also allowed for the detection results to be directly resolved by the naked eye. It could be used as an “off-on” fluorescent probe for the continuous identification of Fe^3+^ and AA. In addition, the intrinsic mechanism of continuous recognition of Fe^3+^ and AA using the RhB@Tb-MOFs was explored in detail. Based on this, the RhB@Tb-MOFs are expected to be a continuous fluorescent sensor for the detection of Fe^3+^ in metal ions and AA in biological small molecules.

## Data Availability

Not applicable.

## Supplementary Materials

The following supporting information can be downloaded at: https://www.mdpi.com/article/10.3390/molecules28155847/s1, Figure S1: (a) Emission spectra of different concentrations of the dye RhB in ethanol; (b) Emission spectra of Tb-MOFs and UV absorption spectra of the RhB. Figure S2: (a) Emission spectra of dye RhB in EtOH solution with different concentrations. (b) Fitted curve between the fluorescent intensity and the RhB of concentration; (c) Emission spectra of RhB@Tb-MOFs containing different concentrations of the RhB; (d) Emission spectra of RhB@Tb-MOFs at different excitation wavelengths (225~245 nm). Figure S3: (a) FT-IR spectra of Tb-MOFs and RhB@ Tb-MOFs; (b) The N2 adsorption isotherms of Tb-MOFs and RhB@Tb-MOFs after heat-treament; (c) The thermogravimetric curve of Tb-MOFs and RhB@Tb-MOFs in the same temperature range; (d) The DTG curve of Tb-MOFs and RhB@Tb-MOFs. Figure S4: (a) SEM images of RhB@Tb-MOFs; (b)~(f) Elemental mapping images of RhB@Tb-MOFs, respectively. Figure S5: (a) SEM image of Tb-MOFs (high magnification), inset shows the details of the sample at this high magnification; (b) SEM image of RhB@Tb-MOFs (high magnification), inset shows the details of the sample at this high magnification. Figure S6: (a) UV absorption spectra of the RhB; (b) UV absorption spectra of Tb-MOFs; (c) UV absorption spectra of RhB@Tb-MOFs. Figure S7: (a) The XPS spectra for Tb-MOFs and RhB@Tb-MOFs; (b) The XPS peaks of Tb 3d in Tb-MOFs; (c) The XPS peaks of Tb 3d in RhB@Tb-MOFs; (d)The XPS peaks of O 1s in Tb-MOFs and RhB@Tb-MOFs; (e) The XPS peaks of O 1s in Tb-MOFs; (f) The XPS peaks of O 1s in RhB@Tb-MOFs. Figure S8: (a) Anti-interference plots of RhB@Tb-MOFs for Fe^3+^ detection in the presence of other interfering metal ions (I_544_); (b) Anti-interference plots of RhB@Tb-MOFs for Fe^3+^ detection in the presence of other interfering metal ions (I_570_); (c) Fluorescence response of Fe^3+^/RhB@Tb-MOFs to various interfering amino acid analytes (10^−3^ M) in the absence and presence of AA. Figure S9: XRD patterns of RhB@Tb-MOFs, Fe^3+^/RhB@Tb-MOFs and AA+Fe^3+^/RhB@Tb-MOFs. Figure S10 XPS spectra of RhB@Tb-MOFs, Fe^3+^/RhB@Tb-MOFs, AA+Fe^3+^/RhB@Tb-MOFs. Table S1. The performance of different probes to detect Fe^3+^. Table S2. The performance of different probes to detect AA. References [12,49,50,51,52,53,54,55,56,57,58,59,60,61,62] are cited in the supplementary materials.

## Sample Availability

Not available.

## References

[1] Guo L., Liu Y., Kong R., Chen G., Liu Z., Qu F., Xia L., Tan W. (2019). A metal–organic framework as selectivity regulator for Fe^3+^ and ascorbic acid detection. Anal. Chem..

[2] Feng D., Zhang T., Zhong T., Zhang C., Tian Y., Wang G. (2021). Coumarin-embedded MOF UiO-66 as a selective and sensitive fluorescent sensor for the recognition and detection of Fe^3+^ ions. J. Mater. Chem. C.

[3] Gao T., Dong B.X., Sun Y., Liu W.L., Teng Y.L. (2019). Fabrication of a water-stable luminescent MOF with an open Lewis basic triazolyl group for the high-performance sensing of acetone and Fe^3+^ ions. J. Mater. Sci..

[4] Nan X., Huyan Y., Li H., Sun S., Xu Y. (2021). Reaction-based fluorescent probes for Hg^2+^, Cu^2+^ and Fe^3+^/Fe^2+^. Coord. Chem. Rev..

[5] Deng L., Zhang Y., Zhang D., Jiao S., Xu J., Liu K., Wang L. (2019). Two exceptionally stable luminescent MOFs for the selective and sensitive detection of Fe^3+^ ions in aqueous solution. CrystEngComm.

[6] Panda S.K., Mishra S., Singh A.K. (2021). Recent progress in the development of MOF-based optical sensors for Fe^3+^. Dalton Trans..

[7] Wu K.-Y., Qin L., Fan C., Cai S.-L., Zhang T.-T., Chen W.-H., Tang X.-Y., Chen J.-X. (2019). Sequential and recyclable sensing of Fe^3+^ and ascorbic acid in water with a terbium(iii)-based metal–organic framework. Dalton Trans..

[8] Suekawa M., Fujikawa Y., Inoue A., Kondo T., Uchida E., Koizumi T., Esaka M. (2019). High levels of expression of multiple enzymes in the Smirnoff-Wheeler pathway are important for high accumulation of ascorbic acid in acerola fruits. Biosci. Biotechnol. Biochem..

[9] Zheng X., Gong M., Zhang Q., Tan H., Li L., Tang Y., Li Z., Peng M., Deng W. (2022). Metabolism and regulation of ascorbic acid in fruits. Plants.

[10] Malik M., Narwal V., Pundir C.S. (2022). Ascorbic acid biosensing methods: A review. Process. Biochem..

[11] Shi X., Li J., Xiong Y., Liu Z., Zhan J., Cai B. (2023). Rh single-atom nanozymes for efficient ascorbic acid oxidation and detection. Nanoscale.

[12] Pirot S.M., Omer K.M., Alshatteri A.H., Ali G.K., Shatery O.B.A. (2023). Dual-template molecularly surface imprinted polymer on fluorescent metal-organic frameworks functionalized with carbon dots for ascorbic acid and uric acid detection. Spectrochim. Acta A.

[13] Yin X., Chen K., Cheng H., Chen X., Feng S., Song Y., Liang L. (2022). Chemical stability of ascorbic acid integrated into commercial products: A review on bioactivity and delivery technology. Antioxidants.

[14] Kong Y., He Y., Zhou J., Zhong S., Song G. (2020). Amino acids as the nitrogen source to synthesize boron nitride quantum dots for fluorescence turn-off-on detection of ascorbic acid. ChemistrySelect.

[15] Dong C.-L., Li M.-F., Yang T., Feng L., Ai Y.-W., Ning Z.-L., Liu M.-J., Lai X., Gao D.-J. (2021). Controllable synthesis of Tb-based metal–organic frameworks as an efficient fluorescent sensor for Cu^2+^ detection. Rare Met..

[16] Li M., Dong C., Yang J., Yang T., Bai F., Ning Z., Gao D., Bi J. (2021). Solvothermal synthesis of La-based metal-organic frameworks and their color-tunable photoluminescence properties. J. Mater. Sci. Mater. Electron..

[17] Sargazi S., Fatima I., Hassan Kiani M., Mohammadzadeh V., Arshad R., Bilal M., Rahdar A., Díez-Pascual A.M., Behzadmehr R. (2022). Fluorescent-based nanosensors for selective detection of a wide range of biological macromolecules: A comprehensive review. Int. J. Biol. Macromol..

[18] Deutchoua A.D.D., Siegnin R., Kouteu G.K., Dedzo G.K., Ngameni E. (2019). Electrochemistry of 2,2-diphenyl-1-picrylhydrazyl (DPPH) in acetonitrile in presence of ascorbic acid—Application for antioxidant properties evaluation. ChemistrySelect.

[19] Zhou X., Qu Q., Wang L., Li L., Li S., Xia K. (2020). Nitrogen dozen carbon quantum dots as one dual function sensing platform for electrochemical and fluorescent detecting ascorbic acid. J. Nanopart. Res..

[20] AbhijnaKrishna R., Velmathi S. (2022). A review on fluorimetric and colorimetric detection of metal ions by chemodosimetric approach 2013–2021. Coord. Chem. Rev..

[21] Amiri M., Haji Shabani A.M., Dadfarnia S., Shokoufi N., Hajipour-Verdom B., Sadjadi S. (2020). Carbon dots doped by nitrogen and sulfur for dual-mode colorimetric and fluorometric determination of Fe^3+^ and histidine and intracellular imaging of Fe^3+^ in living cells. Microchim. Acta.

[22] Khan S., Chen X., Almahri A., Allehyani E.S., Alhumaydhi F.A., Ibrahim M.M., Ali S. (2021). Recent developments in fluorescent and colorimetric chemosensors based on schiff bases for metallic cations detection: A review. J. Environ. Chem. Eng..

[23] Huang X., Yao K., Yu J., Dong W., Zhao Z. (2022). Nitrogen removal performance and microbial characteristics during simultaneous chemical phosphorus removal process using Fe^3+^. Bioresour. Technol..

[24] Sahoo S.K., Crisponi G. (2019). Recent advances on iron(III) selective fluorescent probes with possible applications in bioimaging. Molecules.

[25] Wu Y., Chen Q., Zhao L., Du D., Guo N., Ren H., Liu W. (2020). Spectrofluorometric method for the determination of ascorbic acid in pharmaceutical preparation using l-tyrosine as fluorescence probe. Luminescence.

[26] Hou L., Song Y., Xiao Y., Wu R., Wang L. (2019). ZnMOF-74 responsive fluorescence sensing platform for detection of Fe^3+^. Microchem. J..

[27] Leng J., Lan X., Liu S., Jia W., Cheng W., Cheng J., Liu Z. (2022). Synthesis and bioimaging of a BODIPY-based fluorescence quenching probe for Fe^3+^. Rsc. Adv..

[28] Feng L., Dong C., Li M., Li L., Jiang X., Gao R., Wang R., Zhang L., Ning Z., Gao D. (2020). Terbium-based metal-organic frameworks: Highly selective and fast respond sensor for styrene detection and construction of molecular logic gate. J. Hazard. Mater..

[29] Wong K.-L., Law G.-L., Yang Y.-Y., Wong W.-T. (2006). A highly porous luminescent terbium–organic framework for reversible anion sensing. Adv. Mater..

[30] Majumder S., Chatterjee S., Basnet P., Mukherjee J. (2022). Plasmonic photocatalysis of concentrated industrial LASER dye: Rhodamine 6G. J. Mol. Liq..

[31] Ruan B., Yang J., Zhang Y.-J., Ma N., Shi D., Jiang T., Tsai F.-C. (2020). UiO-66 derivate as a fluorescent probe for Fe^3+^ detection. Talanta.

[32] Li M., Gao Y., Yang W., Zhang C., Fang Y., Wang C., Song S., Pan Q. (2022). Dye-encapsulated lanthanide-based metal–organic frameworks as a dual-emission sensitization platform for alachlor sensing. Inorg. Chem..

[33] Wang H., Yang T., Feng L., Ning Z., Liu M., Lai X., Gao D., Bi J. (2018). Energy transfer and multicolor tunable luminescence properties of NaGd_0.5_Tb_0.5−x_Eu_x_(MoO_4_)_2_ phosphors for UV-LED. J. Electron. Mater..

[34] Wu S., Zhu M., Zhang Y., Kosinova M., Fedin V.P., Gao E. (2021). Luminescent sensors based on coordination polymers with adjustable emissions for detecting biomarker of pollutant ethylbenzene and styrene. Appl. Organomet. Chem..

[35] Wu S., Zhu M., Zhang Y., Kosinova M., Fedin V.P., Gao E. (2020). A water-stable lanthanide coordination polymer as multicenter platform for ratiometric luminescent sensing antibiotics. Chem. Eur. J..

[36] Gong M., Li Z., Wang Q., Xiang W., Xia T., Zhao D. (2022). Encapsulating rhodamine B in the NbO-type metal-organic framework to construct dual-emitting ratiometric thermometer. J. Solid State Chem..

[37] Sánchez F., Gutiérrez M., Douhal A. (2022). Novel approach for detecting vapors of acids and bases with proton-pransfer luminescent dyes encapsulated within metal-organic frameworks. ACS Appl. Mater. Inter..

[38] Che J., Jiang X., Fan Y., Li M., Zhang X., Gao D., Ning Z., Li H. (2022). A novel dual-emission fluorescence probe based on CDs and Eu^3+^ functionalized UiO-66-(COOH)_2_ hybrid for visual monitoring of Cu^2+^. Materials.

[39] Jin Y., Yan B. (2021). A bi-functionalized metal-organic framework based on N-methylation and Eu^3+^ post-synthetic modification for highly sensitive detection of 4-Aminophenol (4-AP), a biomarker for aniline in urine. Talanta.

[40] Fan Y., Jiang X., Che J., Li M., Zhang X., Gao D., Bi J., Ning Z. (2022). A ratiometric fluorescent sensor based on Dye/Tb (III) functionalized UiO-66 for highly sensitive detection of TDGA. Molecules.

[41] Liu J., Ye L.Y., Mo Y.Y., Yang H. (2022). Highly sensitive fluorescent quantification of acid phosphatase activity and its inhibitor pesticide Dufulin by a functional metal–organic framework nanosensor for environment assessment and food safety. Food Chem..

[42] Hao J.-N., Yan B. (2016). A dual-emitting 4d–4f nanocrystalline metal–organic framework as a self-calibrating luminescent sensor for indoor formaldehyde pollution. Nanoscale.

[43] Xu X.-Y., Yan B. (2015). Eu(III)-functionalized MIL-124 as fluorescent probe for highly selectively sensing iIons and organic small molecules especially for Fe(III) and Fe(II). ACS Appl. Mater. Inter..

[44] Li Y., Ling H.-X., Gao Y., Zhang S., Yan B. (2022). Lanthanide β-diketonate complex functionalized poly(ionic liquid)s/SiO_2_ microsphere as a fluorescent probe for the determination of bovine hemoglobin. ACS Appl. Polym. Mater..

[45] He Q., Zhuang S., Yu Y., Li H., Liu Y. (2021). Ratiometric dual-emission of Rhodamine-B grafted carbon dots for full-range solvent components detection. Anal. Chim. Acta.

[46] Li S., Liu T., Yan B. (2022). Dye functionalized lanthanide metal–organic framework as a multifunctional luminescent hybrid material for visual sensing of biomarker 2-methoxyaceticacid and sulfide anion. J. Colloid Interface Sci..

[47] Dalapati R., Biswas S. (2019). Aqueous phase sensing of Fe^3+^ and ascorbic acid by a metal–organic framework and its implication in the construction of multiple logic gates. Chem. Asian J..

[48] Yang J., Che J., Jiang X., Fan Y., Gao D., Bi J., Ning Z. (2022). A novel turn-on fluorescence probe based on Cu(II) functionalized metal–organic frameworks for visual detection of uric acid. Molecules.

[49] Sun J., Hong Y.-L., Fang X.-Q., Wang C., Liu C.-M. (2023). Fluorescent phosphine oxide-containing hyperbranched polyesters: Design, synthesis and their application for Fe^3+^ detection. J. Mater. Chem. C.

[50] Pang S., Liu S. (2020). Dual-emission carbon dots for ratiometric detection of Fe^3+^ ions and acid phosphatase. Anal. Chim. Acta.

[51] Cui J., Zhu X., Liu Y., Liang L., Peng Y., Wu S., Zhao Y. (2022). N-Doped Carbon Dots as Fluorescent “Turn-Off” Nanosensors for Ascorbic Acid and Fe^3+^ Detection. ACS Appl. Nano Mater..

[52] Geng R., Tang H., Ma Q., Liu L., Feng W., Zhang Z. (2022). Bimetallic Ag/Zn-ZIF-8: An efficient and sensitive probe for Fe^3+^ and Cu2+ detection. Colloid Surf. A.

[53] Zhang T., Salah A., Chang S., Zhang Z., Wang G. (2021). Study on the fluorescent covalent organic framework for selective “turn-off”recognition and detection of Fe^3+^ ions. Tetrahedron.

[54] Xu H., Dong Y., Wu Y., Ren W., Zhao T., Wang S., Gao J. (2018). An -OH group functionalized MOF for ratiometric Fe^3+^ sensing. J. Solid State Chem..

[55] Yang X., Liang Y., Feng W., Yang C., Wang L., Huang G., Wang D. (2022). Hollow terbium metal–organic-framework spheres: Preparation and their performance in Fe^3+^ detection. RSC Adv..

[56] Zhang X., Feng L., Ma S., Xia T., Jiao F., Kong Z., Duan X. (2022). A microporous Tb-based MOF for multifunctional detection of the α-CHC, Cu^2+^ and Fe^3+^. J. Solid State Chem..

[57] Xiao J., Liu J., Liu M., Ji G., Liu Z. (2019). Fabrication of a Luminescence-Silent System Based on a Post-Synthetic Modification Cd-MOFs: A Highly Selective and Sensitive Turn-on Luminescent Probe for Ascorbic Acid Detection. Inorg. Chem..

[58] Wan H., Wang Y., Chen J., Meng H.-M., Li Z. (2021). 2D Co-MOF nanosheet-based nanozyme with ultrahigh peroxidase catalytic activity for detection of biomolecules in human serum samples. Microchim. Acta.

[59] Li M., Zhang S., Li H., Chen M. (2023). Cerium/polyacrylic acid modified porphyrin metal-organic framework as fluorescence and photothermal sensor for ascorbic acid measurement. Talanta.

[60] Wang X., Long C., Jiang Z., Qing T., Zhang K., Zhang P., Feng B. (2019). In situ synthesis of fluorescent copper nanoclusters for rapid detection of ascorbic acid in biological samples. Anal. Methods-UK.

[61] Sun M., Zhong Z., Wang Y., Yu B., Zhang L., Zhang W. (2023). Dual-functional lanthanide-MOF probe nanocomposite based on hydroxyapatite nanowires as fluorescent sensor for ascorbic acid. Microchim. Acta.

[62] Selivanova N., Galyametdinov Y. (2021). Terbium(III) as a Fluorescent Probe for Molecular Detection of Ascorbic Acid. Chemosensors.

