# Supplementary material for: A Ratiometric Fluorescent Probe Based on RhB Functionalized Tb-MOFs for the Continuous Visual Detection of Fe3+ and AA
Source: Molecules. 2023 Aug 3;28(15):5847. doi: 10.3390/molecules28155847 (PMC10421046; doi:10.3390/molecules28155847)
Supplement: Supplementary file 1 [file molecules-28-05847-s001.zip › molecules-2497089-supplementary.pdf]

Supporting Information

Characterization of the RhB@Tb-MOFs composites

The fluorescence emission spectra of RhB@Tb-MOFs samples phosphors were obtained on a box cell filled with dry powders by using a F-7000 fluorescence spectrophotometer (Hitachi, Japan) equipped with a 150 W Xenon lamp as the excitation source. The scanning speed tested was 1200 nm/min. Powder X-ray diffraction (XRD) measurements were performed on a Smart Lab powder diffractometer (Rigaku, Japan) with Cu Kα radiation (λ = 0.15406 nm) at a scanning rate of 8°/min in the 2θ range from 5° to 75°. The microstructures of all samples were examined on a Quanta 250 scanning electron microscope (FEI, America). Photoluminescence decay curves were measured by Steady/Transient State Fluorescence Spectrometer, F900, Edinburgh Instruments, UK. All measurements were performed at room temperature.

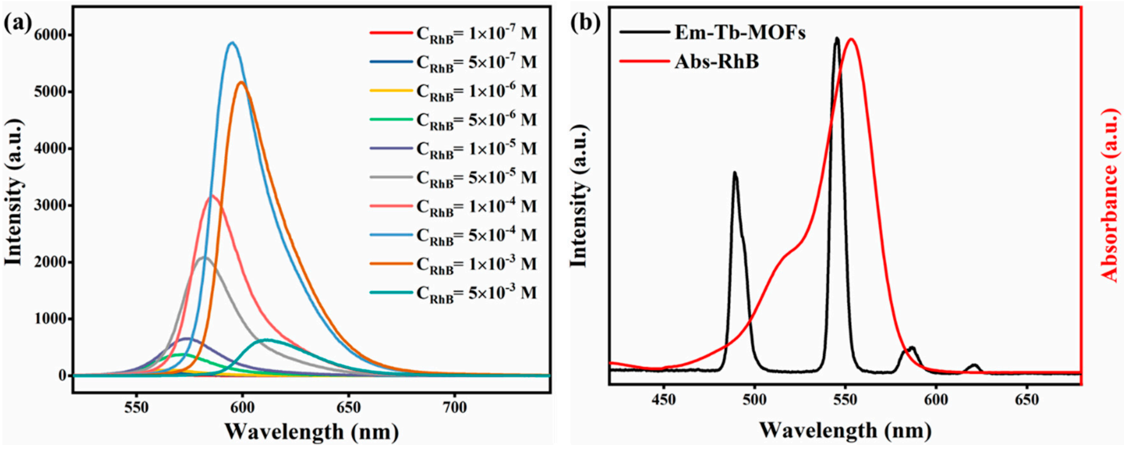

Figure S1 (a) Emission spectra of different concentrations of the dye RhB in ethanol; (b) Emission spectra of Tb-MOFs and UV absorption spectra of the RhB.

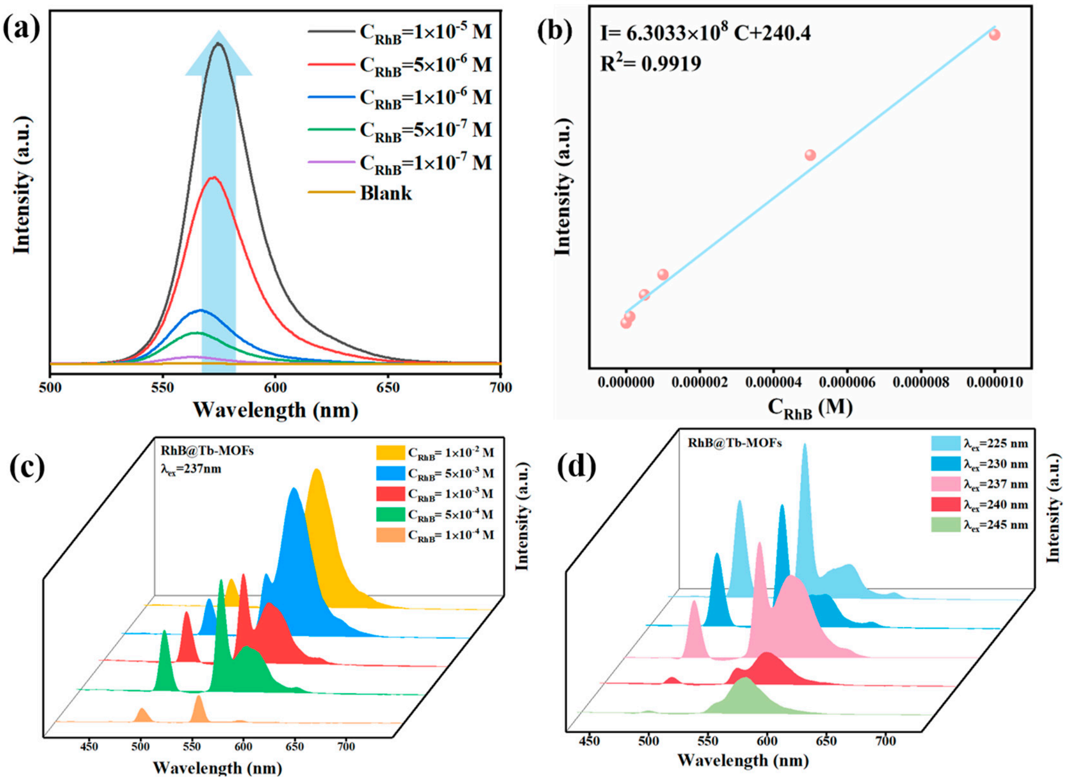

Figure S2 (a) Emission spectra of dye RhB in EtOH solution with different concentrations. (b) Fitted curve between the fluorescent intensity and the RhB of concentration; (c) Emission spectra of RhB@Tb-MOFs containing different concentrations of the RhB; (d) Emission spectra of RhB@Tb-MOFs at different excitation wavelengths (225~245 nm).

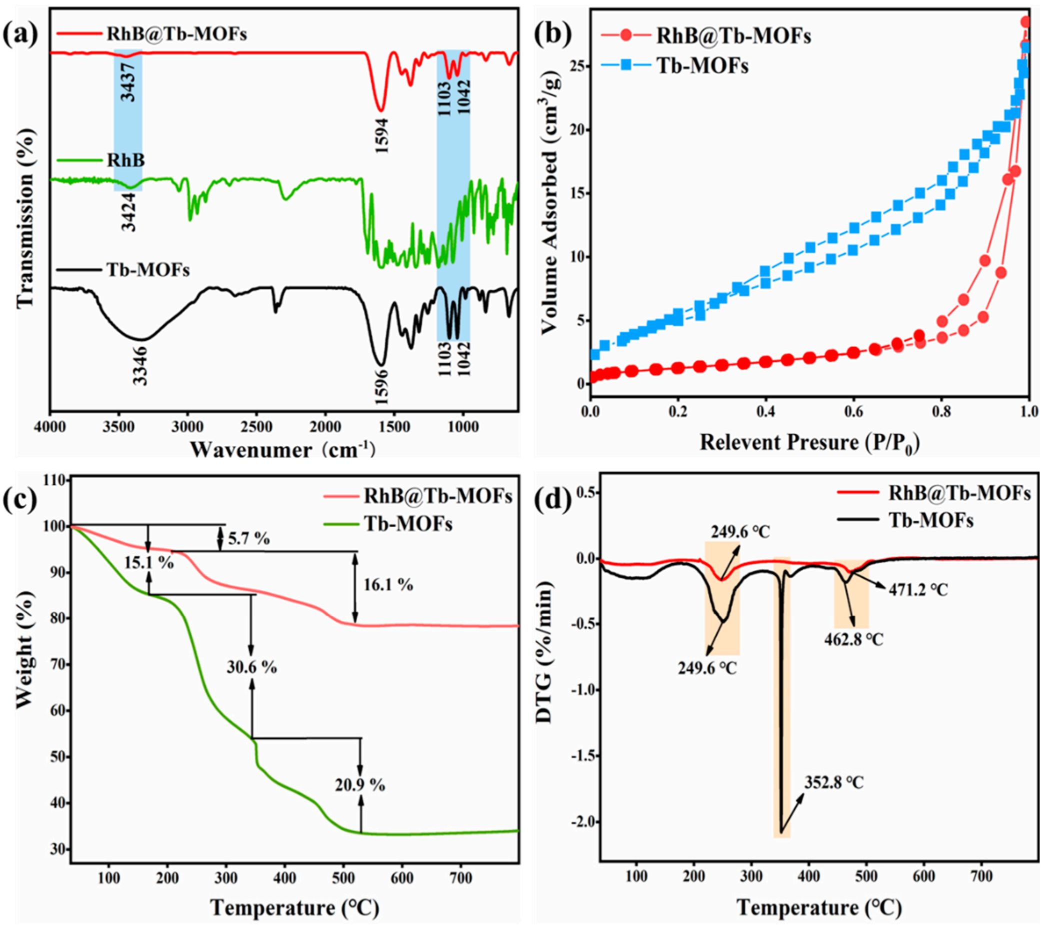

Figure S3 (a) FT-IR spectra of Tb-MOFs and RhB@ Tb-MOFs; (b) The N_2 adsorption isotherms of Tb-MOFs and RhB@Tb-MOFs after heat-treament; (c) The thermogravimetric curve of Tb-MOFs and RhB@Tb-MOFs in the same temperature range; (d) The DTG curve of Tb-MOFs and RhB@Tb-MOFs.

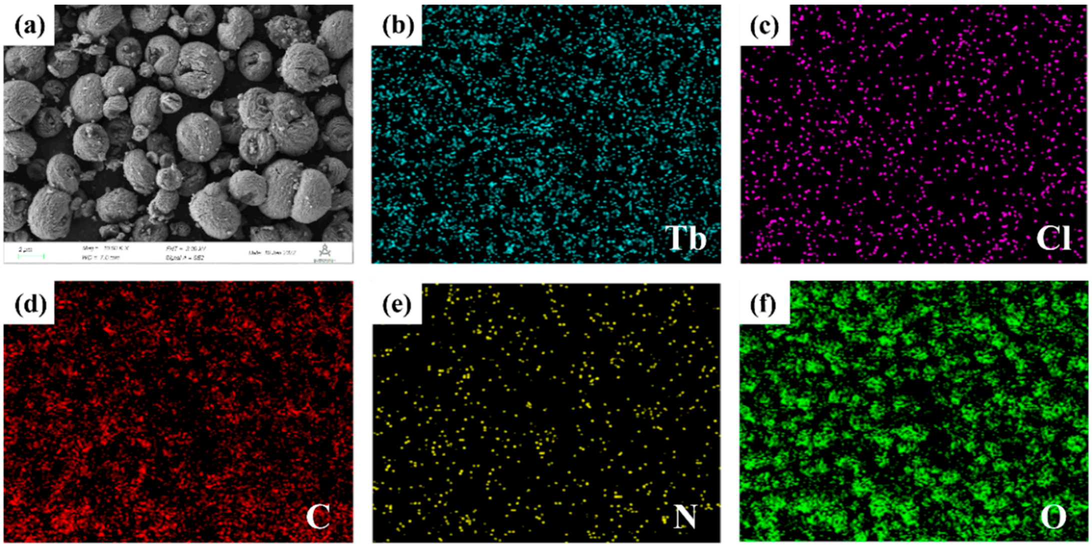

Figure S4 (a) SEM images of RhB@Tb-MOFs; (b)~(f) Elemental mapping images of RhB@Tb-MOFs, respectively.

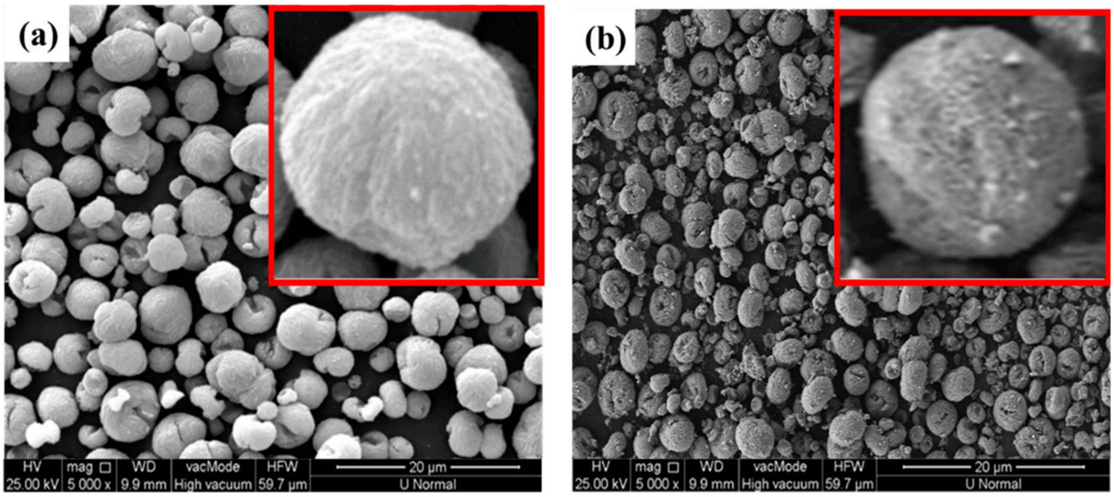

Figure S5 (a) SEM image of Tb-MOFs (high magnification), inset shows the details of the sample at this high magnification; (b) SEM image of RhB@Tb-MOFs (high magnification), inset shows the details of the sample at this high magnification.

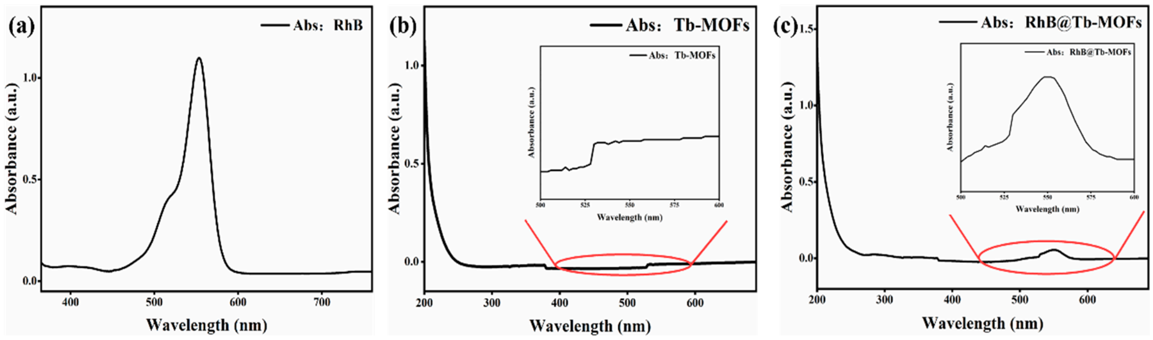

Figure S6 (a) UV absorption spectra of the RhB; (b) UV absorption spectra of Tb-MOFs; (c) UV absorption spectra of RhB@Tb-MOFs.

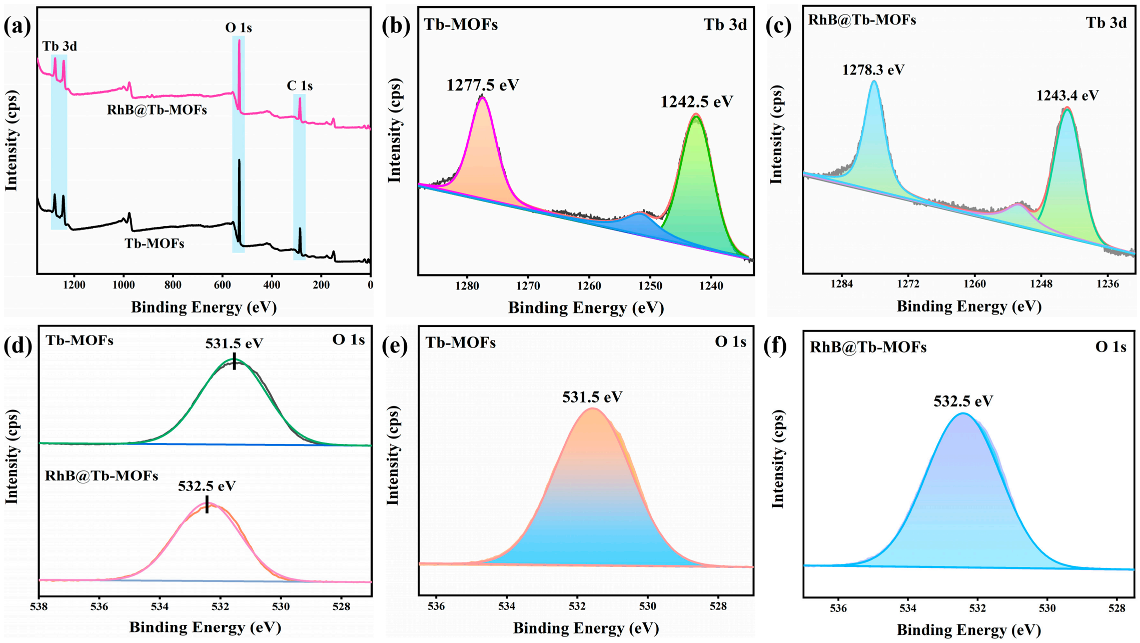

Figure S7 (a) The XPS spectra for Tb-MOFs and RhB@Tb-MOFs; (b) The XPS peaks of Tb 3d in Tb-MOFs; (c) The XPS peaks of Tb 3d in RhB@Tb-MOFs; (d)The XPS peaks of O 1s in Tb-MOFs and RhB@Tb-MOFs; (e) The XPS peaks of O 1s in Tb-MOFs; (f) The XPS peaks of O 1s in RhB@Tb-MOFs.

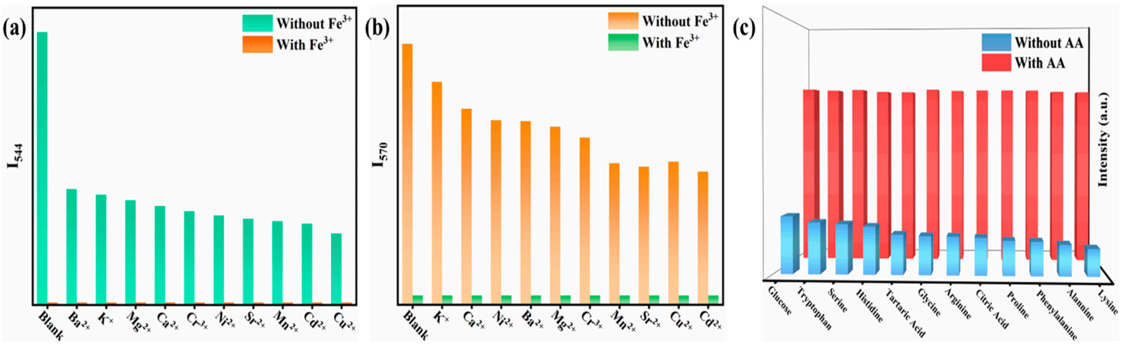

Figure S8 (a) Anti-interference plots of RhB@Tb-MOFs for Fe^{3+} detection in the presence of other interfering metal ions (I_{544}); (b) Anti-interference plots of RhB@Tb-MOFs for Fe^{3+} detection in the presence of other interfering metal ions (I_{570}); (c) Fluorescence response of Fe^{3+}/RhB@Tb-MOFs to various interfering amino acid analytes (10^{-3} M) in the absence and presence of AA.

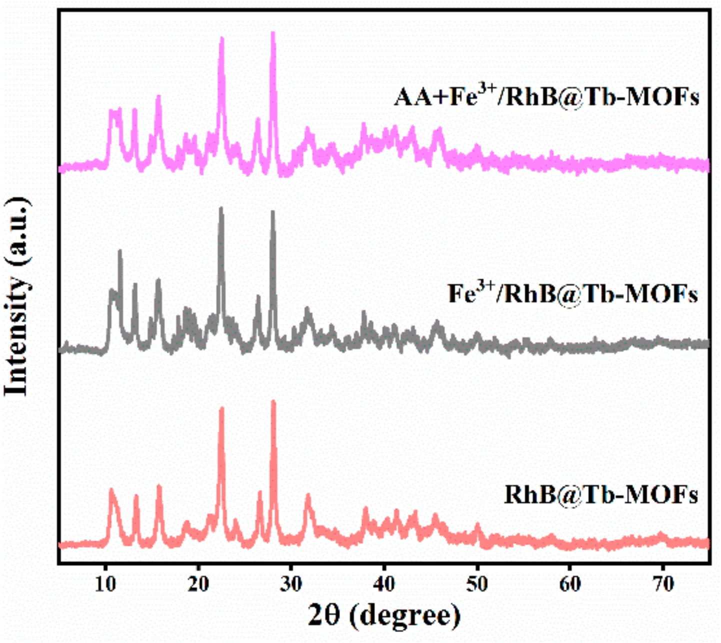

Figure S9 XRD patterns of RhB@Tb-MOFs, Fe^{3+}/RhB@Tb-MOFs and AA+Fe^{3+}/RhB@Tb-MOFs.

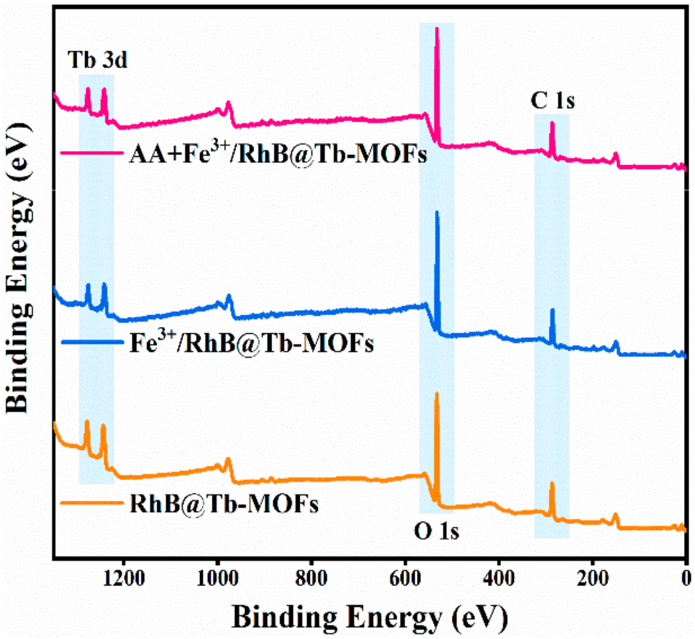

Figure S10 XPS spectra of RhB@Tb-MOFs, Fe^{3+}/RhB@Tb-MOFs, AA+Fe^{3+}/RhB@Tb-MOFs.

Table S1. The performance of different probes to detect Fe^{3+}.

Probe	Analyte	Detection range	LOD	Reference
HBP2	Fe^{3+}	0-1000 μM	0.59 μM	[1]
GN-CDs	Fe^{3+}	0-50 μM	0.8 μM	[2]
NCDs	Fe^{3+}	5-60 μM	1.9 μM	[3]
Ag/Zn-ZIF-8	Fe^{3+}	1-10 μM	3.9 μM	[4]
TT-COF	Fe^{3+}	2-20 μM	84 μM	[5]
$Eu_2(OH\text{-}BDC)_3$	Fe^{3+}	10-50 μM	1.17 μM	[6]
Tb-MOFs	Fe^{3+}	0-10 μM	2.05 μM	[7]
Tb-TATB	Fe^{3+}	0-200 μM	4.84 μM	[8]
RhB@Tb-MOFs	Fe^{3+}	10-10000 μM	0.47 μM	This work

Table S2. The performance of different probes to detect AA

Probe	Analyte	Detection range	R^2	LOD	Reference
CrO_4^{2-}@Cd-MOFs	AA	46.3-591 μM	0.988	7.27 ppm	[9]
2D Co-MOF	AA	0-16 μM	0.9941	0.47 μM	[10]
PCN-224@PAA-Ce	AA	3-80 μM	0.9898	1.4 μM	[11]
CuNCs	AA	501-000 μM	0.997	41.94 μM	[12]
Tb-MOF	AA	0.3-40 μM	0.9921	20.4 nM	[13]
Tb(1,10-phenanthroline)	AA	1.6-380 μM	0.9984	74 μM	[14]
Cr-MOF	AA	25.0-425.0 μM	0.996	1.10 μM	[15]
Fe^{3+}/RhB@Tb-MOFs	AA	10-100 μM	0.9965	2.54 μM	This work

Reference

1. Sun, J.; Hong, Y.-L.; Fang, X.-Q.; Wang, C.; Liu, C.-M., Fluorescent phosphine oxide-containing hyperbranched polyesters: design, synthesis and their application for Fe^{3+} detection. *J Mater Chem C C* **2023**, 11, (5), 1927-1936.
2. Pang, S.; Liu, S., Dual-emission carbon dots for ratiometric detection of Fe^{3+} ions and acid phosphatase. *Anal Chim Act*a **2020**, 1105, 155-161.
3. Cui, J.; Zhu, X.; Liu, Y.; Liang, L.; Peng, Y.; Wu, S.; Zhao, Y., N-Doped Carbon Dots as Fluorescent "Turn-Off" Nanosensors for Ascorbic Acid and Fe^{3+} Detection. *Acs Appl Nano Mater* **2022**, 5, (5), 7268-7277.
4. Geng, R.; Tang, H.; Ma, Q.; Liu, L.; Feng, W.; Zhang, Z., Bimetallic Ag/Zn-ZIF-8: An efficient and sensitive probe for Fe^{3+} and Cu^{2+} detection. *Colloid Surface A* **2022**, 632, 127755.
5. Zhang, T.; Salah, A.; Chang, S.; Zhang, Z.; Wang, G., Study on the fluorescent covalent organic framework for selective "turn-off"recognition and detection of Fe^{3+} ions. *Tetrahedron* **2021**, 96, 132405.
6. Xu, H.; Dong, Y.; Wu, Y.; Ren, W.; Zhao, T.; Wang, S.; Gao, J., An -OH group functionalized MOF for ratiometric Fe^{3+} sensing. *J Solid State Chem* **2018**, 258, 441-446.
7. Yang, X.; Liang, Y.; Feng, W.; Yang, C.; Wang, L.; Huang, G.; Wang, D., Hollow terbium metal–organic-framework spheres: preparation and their performance in Fe^{3+} detection. *Rsc Adv* **2022**, 12, (7), 4153-4161.
8. Zhang, X.; Feng, L.; Ma, S.; Xia, T.; Jiao, F.; Kong, Z.; Duan, X., A microporous Tb-based MOF for multifunctional detection of the α-CHC, Cu^{2+} and Fe^{3+}. *J Solid State Chem* **2022**, 312, 123232.
9. Xiao, J.; Liu, J.; Liu, M.; Ji, G.; Liu, Z., Fabrication of a Luminescence-Silent System Based on a Post-Synthetic Modification Cd-MOFs: A Highly Selective and Sensitive Turn-on Luminescent Probe for Ascorbic Acid Detection. *Inorg Chem* **2019**, 58, (9), 6167-6174.
10. Wan, H.; Wang, Y.; Chen, J.; Meng, H.-M.; Li, Z., 2D Co-MOF nanosheet-based nanozyme with ultrahigh peroxidase catalytic activity for detection of biomolecules in human serum samples. *Microchim Acta* **2021**, 188, (4), 130.
11. Li, M.; Zhang, S.; Li, H.; Chen, M., Cerium/polyacrylic acid modified porphyrin metal-organic framework as fluorescence and photothermal sensor for ascorbic acid measurement. *Talanta* **2023**, 252, 123825.
12. Wang, X.; Long, C.; Jiang, Z.; Qing, T.; Zhang, K.; Zhang, P.; Feng, B., In situ synthesis of fluorescent copper nanoclusters for rapid detection of ascorbic acid in biological samples. *Anal Methods-Uk* **2019**, 11, (36), 4580-4585.
13. Sun, M.; Zhong, Z.; Wang, Y.; Yu, B.; Zhang, L.; Zhang, W., Dual-functional lanthanide-MOF probe nanocomposite based on hydroxyapatite nanowires as fluorescent sensor for ascorbic acid. *Microchim Acta* **2023**, 190, (3), 89.
14. Selivanova, N.; Galyametdinov, Y., Terbium(III) as a Fluorescent Probe for Molecular Detection of Ascorbic Acid. *Chemosensors* **2021**, 9, 134.
15. Pirot, S. M.; Omer, K. M.; Alshatteri, A. H.; Ali, G. K.; Shatery, O. B. A., Dual-template molecularly surface imprinted polymer on fluorescent metal-organic frameworks functionalized with carbon dots for ascorbic acid and uric acid detection. *Spectrochim Acta A* **2023**, 291, 122340.
